# Ankylosing Spondylitis Is Associated With Risk of New-Onset Obstructive Sleep Apnea: A Nationwide Population-Based Cohort Study

**DOI:** 10.3389/fmed.2019.00285

**Published:** 2019-12-06

**Authors:** Chien-Han Tsao, Jing-Yang Huang, Hsin-Hsin Huang, Yao-Min Hung, James Cheng-Chung Wei, Yin-Tsan Hung

**Affiliations:** ^1^Department of Otolaryngology, Chung Shan Medical University Hospital, Taichung, Taiwan; ^2^School of Medicine, Chung Shan Medical University, Taichung, Taiwan; ^3^Department of Medical Research, Chung Shan Medical University Hospital, Taichung, Taiwan; ^4^Institute of Medicine, Chung Shan Medical University, Taichung, Taiwan; ^5^Yuh-Ing Junior College of Health Care and Management, Kaohsiung, Taiwan; ^6^School of Medicine, National Yang-Ming University, Taipei, Taiwan; ^7^Department of Internal Medicine, Kaohsiung Municipal United Hospital, Kaohsiung, Taiwan; ^8^Division of Allergy, Immunology and Rheumatology, Chung Shan Medical University Hospital, Taichung, Taiwan; ^9^Graduate Institute of Integrated Medicine, China Medical University, Taichung, Taiwan; ^10^Department of Otolaryngology, BenQ Medical Center, The Affiliated BenQ Hospital of Nanjing Medical University, Nanjing, China

**Keywords:** ankylosing spondylitis, obstructive sleep apnea, population-based cohort study, risk, medicine

## Abstract

**Objectives:** Investigating the risk of obstructive sleep apnea(OSA) among ankylosing spondylitis (AS) patients based on administrative healthcare databases.

**Methods:** We conducted a nationwide cohort study by using the Taiwan National Health Insurance Research Database with 1997–2013 claim records. The AS cohort included 2,210 patients who were newly diagnosed between 2003 and 2013. Randomly selected non-AS controls were matched at a 1:4 ratio. The endpoint was set as OSA occurrence or the end of 2013. Cumulative incidences, hazard ratios (HRs) and 95% confidence intervals (CIs) were calculated after adjusting for age, gender, comorbidities, and co-medication. Multivariate analyses were performed using the Cox proportional hazards model. Due to the violation of the proportionality assumption, landmark analysis was conducted to explore the risk of OSA during specific follow-up periods.

**Results:** The adjusted HR (aHR) of OSA in the AS group was 2.826 (95% C.I. = 1.727–4.625) compared to the control group. On landmark analysis, aHR was 7.919 (95% C.I. = 3.169–19.792) for the AS group 0–24 months from the index date and decreased to 1.816 (95% C.I. = 0.944–3.494) at ≥ 24 months from the index date. The increased risks of OSA in the AS group compared to the control group were found for both males and females (aHRs were 4.533 and 2.672). On age-stratified analysis, a significant risk only for the 40–59 age group with aHR of 3.913 (95% C.I. = 1.890–8.102).

**Conclusions:** A higher risk of developing OSA was found among newly diagnosed AS cohort during the maximum 11-year follow-up period, especially within 2 years after newly diagnosed AS and in the 40–59 age group.

## Key Points

This study delved into a possible association about patients with newly diagnosed ankylosing spondylitis (AS) and higher risk of developing obstructive sleep apnea (OSA).

1. We demonstrated a 2.8-fold risk of development OSA among AS patients than the general population.
2. Stratified analyses revealed significant effects for both genders and the 40–59 age group.
3. Developing OSA was noted in the first 2 years after diagnosis of AS (aHR was 7.919 with 95% C.I. = 3.169–19.792).

Clinically, physicians and patients should be aware of this possible association.

## Introduction

Obstructive sleep apnea (OSA) is a common chronic disorder characterized by recurrent collapse of the upper airway during sleep, leading to sleep fragmentation, and daytime sleepiness (1–3). Individuals with OSA present with apneas, hypopneas, or respiratory effort-related arousals, occurring at least 5 times/h during sleep (apnea–hypopnea index, AHI ≥5) (2, 3). The estimated prevalence in North America is about 20–30 percent in males and 10–15 percent in females when OSA is defined broadly as an AHI >5 events per hour as measured on polysomnogram (4, 5). Even with the more stringent definition of AHI ≥15 events per hour, the estimated prevalence is around 15 percent in males and 5 percent in females (5, 6). A recent population-based study demonstrated a need to revise the definition of this disease and presented high prevalence rates for moderate to severe OSA (AHI≥15) (23.4% in women and 49.7% in men) (7). Even though OSA is not an immediate life-threatening disease, it can lower quality of life and productivity, increase risk of hospitalization and elevate morbidity from cardiovascular diseases (8–10). There have been several studies showing that patients with OSA have higher healthcare service utilization, including medical costs, medication usage, emergency department visits, and hospitalization compared to subjects without OSA in the US (11, 12), Canada (13), Denmark (14), Israel (15), and Taiwan (16).

Well-defined risk factors for OSA include age, male gender, obesity, and upper airway soft tissue abnormalities. Potential risk factors include smoking, nasal congestion, and family history (17–19). Systemic autoimmune diseases are characterized by dysregulation of the immune system, which in turn activates the immune cells to attack autoantigens resulting in inappropriate inflammation and multi-tissue damage. OSA has been linked to inflammation, coagulation and endothelial dysfunction (20). Therefore, the correlation between autoimmune diseases and OSA deserves attention. The results of a previous study have shown an association between autoimmune disease as:rheumatoid arthritis (RA), primary Sjogren syndrome, SLE, bechet disease and subsequent OSA (21–23). However, the relation of OSA to single autoimmune disease, ankylosing Spondylitis(AS), which was not been discussed in this single race longitudinal nationwide cohort study.

AS is a chronic inflammatory disease, a type of spondyloarthritis characterized by spondylitis, sacroilitis, peripheral joint involvement, and enthesitis (24). The prevalence of As is associated to the genetic factor, the MHC class molecule HLA B27 in population (25). In addition to affecting the musculoskeletal system, AS exhibits a wide range of extra-articular manifestations, in respiratory system: such as interstitial lung disease (ILD) (26), in gastro-intestine system; such as inflammatory bowel disease (IBD), Crohn's disease, in cardiovascular system; such as atherosclerosis and atherosclerotic CVD, valvular heart disease (27), arrhythmias, in ophthalmogy and dermatology were psoriasis and uvitis, asthma, and OSA (22, 28–33).

The prevalence of OSA in AS patients is higher than that reported in the general population, but it is not easy to identify OSA in AS patients if no detailed testing of polysomnography (29, 33, 34). A recent population study showed the aHR:3.76 of OSAS between AS to general population without long term follow up (31) and the previous studies have been performed with small study populations or cross sectional studies without long term follow up (29). Therefore, they cannot be used to explain the temporal relationship between AS and OSA. Due to a lack of research on the epidemiological relationship between AS and the subsequent development of OSA, this longitudinal nationwide cohort study was conducted to explore whether patients with newly diagnosed AS are prone to the subsequent development of OSA. Our study presented the relation of OSA to single autoimmune disease, Ankylosing Spondylitis, which was not been discussed in a single race longitudinal nationwide cohort study.

## Materials and Methods

### Data Source

The Longitudinal Health Insurance Research Datasets (LHIRD) were collected from the National Health Insurance (NHI) program, which is a single-payer, social insurance system, covering 94% of the population in 2000. The randomly sampled beneficiaries (*n* = 1 million) of LHIRD were registered in the NHI program in 2000. The 1997–2013 claim datasets, including outpatient visits, discharge records, and prescription data of LHIRD were retrieved for analysis. Identifiers were scrambled to protect the privacy of subjects. This study was approved by the Institutional Review Board of Chung Shan Medical University in Taiwan (IRB permit number CS15134), which waived the requirement for informed consent due to the anonymous use of data with subjects unidentifiable before analysis.

### Patients With Ankylosing Spondylitis (AS)

This retrospective cohort study was conducted using administrative claims records. Patients with AS (ICD-9 code: 720.0), as defined by the 1984 modified New York criteria (Please deleted) were identified based on at least 2 outpatient visits or 1 admission within 1 year by rheumatologist, orthopedist, or rehabilitation physician. There were 4,990 AS patients and 917,042 non-AS individuals from 1997–2013 included in the LHIRD. In order to observe the risk of OSA from new-onset AS, we excluded cases with AS before 2003 (*n* = 2,086). Furthermore, we excluded AS patients who did not receive spinal X-ray within 6 months before or after AS diagnosis (*n* = 672), or with OSA event before AS diagnosis (*n* = 22). Finally, there were 2,210 AS patients newly diagnosed with AS from 2003 to 2013, and the index date was the first date of AS diagnosis.

The 1:4 age-sex individual matched controls were randomly sampled from among the non-AS individuals. The index date for the controls corresponded to the date of matched AS case. All study participants met the inclusion criteria and were at risk at index date.

### Identified Patients of Obstructive Sleep Apnea (OSA)

Newly diagnosed OSA (ICD-9 code: 327.23, 780.51, 780.53, and 780.57) was identified from index date of newly diagnosed AS to the end of the study (Dec 2013) or withdrawal from the NHI program.

In order to increase the validity of diagnosis of OSA, we sued the following 2 criteria: (1) ICD-9 code: 327.23, 780.51, 780.53 and 780.57 (2) OSA diagnoses made by otolaryngologist, neurologist, or chest physician and patients must fit non-rule out outpatient visit ≥ 2 times or at least 1 inpatients admission. We only considered OSA diagnoses made by otolaryngologist, neurologist, or chest physician. Due to the result of polysomnography were unavailable in the NHIRD database, and non-OSA (AHI<5) people may be mis-arranged to OSA group. The option of arranging polysomnography or not was abandon form criteria. There were 30 (1.36%) and 40 (0.45%) OSA cases diagnosed by otolaryngologist, neurologist, or chest physician in AS and non-AS groups, respectively.

### Confounding Comorbidities and Co-medications

The comorbidities analyzed in this study were hypertension (ICD-9-CM codes 401–405), diabetes mellitus (ICD-9-CM code 250), hyperlipidemia (ICD-9-CM code 272), chronic obstructive pulmonary disease (COPD) (ICD-9-CM codes 491, 492, 496), asthma (ICD-9-CM code 493), cancer (ICD-9-CM codes 140–208), chronic liver diseases (ICD-9-CM code 571.4), hepatitis B (ICD-9-CM codes 070.2, 070.3, V02.61), hepatitis C (ICD-9-CM codes 070.41, 070.44, 070.51, 070.54, V02.62), coronary artery disease (CAD) (ICD-9-CM codes 410–414), dysrhythmia (ICD-9-CM code 427), congestive heart failure (CHF) (ICD-9-CM code 428), stroke (ICD-9-CM codes 430–438), chronic kidney disease (CKD) (ICD-9-CM code 585), asthma (ICD-9-CM codes 493), thyroid disorders (ICD-9: 240, 241, 242, 244.9, 245.0, 245.1, 245.2), other rheumatic diseases (ICD-9: 714, 710, 696.0, 696.1), RA (ICD-9 code: 714.0), systemic lupus erythematosus (SLE) (ICD-9 code: 710.0), Sjögren's syndrome (ICD-9 code: 710.2) and psoriasis (ICD-9 code: 696.0, 696.1), Other respiratory abnormalities(ICD-9 code: 786.09) which covered much symptoms over snoring, other form of dyspnea, other abnormalities of breathing, and hypertrophic tonsillitis (ICD-9: 474.10) for upper airway soft tissue abnormalities. The associated cord for nasal congestion including: adenoid hypertrophy (ICD-9: 474.12), chronic rhinitis (ICD-9: 472.0), hypertrophy of nasal turbinate (ICD-9: 478) and deviated for nasal septum (ICD-9: 470). Information on comorbid medical disorders was obtained by tracing all ambulatory medical care and inpatient records in the NHI database within 2 years of the index visit. The medication confounders in this study were corticosteroids, non-steroidal anti-inflammatory drugs (NSAIDs), proton pump inhibitors (PPIs), H_2_ receptor antagonists, aspirin, oral antihypertensive drugs (including alpha-blockers, beta- blockers, angiotensin-converting enzyme inhibitors (ACEIs), angiotensin receptor blockers (ARBs), and calcium channel blockers (CCBs), oral hypoglycemic agents (including biguanides, sulfonylureas, alpha glucosidase inhibitors, thiazolidinediones), and statins. Drug use was defined as usage of that drug for ≥ 30 days within 180 days before and after index date.

### Statistical Analysis

The chi-square test was used to test the homogeneity of category variables between AS and control groups. After examining the proportional hazard assumption, the risk of OSA from AS exposure was found to be time dependent (**Figure 2**). Therefore, landmark analysis was performed to analyze the OSA risk during 2 specific time intervals(index date to 24 and ≥24 months after index date). All study individuals were followed from index date occurrence of earliest of the following: occurrence of OSA, death, or end of study (DEC 2013). Univariate and multivariate Cox regression models we reused to estimate the crude and adjusted hazard ratios (HRs, 95% confidence interval, 95% C.I.). Furthermore, subgroup analysis was used to explore the interaction factors. All statistical analyses were performed with SAS software (version 9.4; SAS Institute, Cary, NC, USA). A *p* < 0.05 indicated statistical significance.

## Results

**Figure 1:**
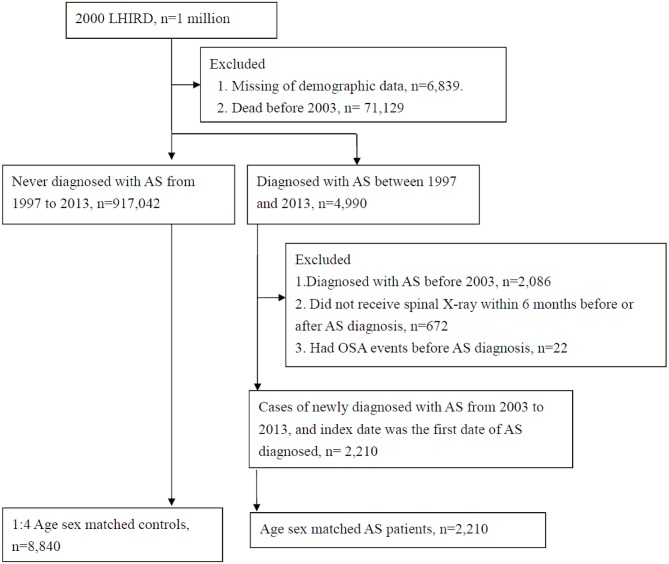
Flow chart of subjects selection.

After applying the inclusion and exclusion criteria and carrying out age-sex matching, 2,210 AS patients and 8,840 controls were enrolled (Figure 1). Table 1 provides the baseline characteristics of the study groups. Among AS patients, 79.14% were 20–59 years old and 64.62% were male. There were significantly lower proportions of low-income households, longer hospital stays, higher proportions of co-morbidities (such as other rheumatic diseases, thyroid disorders, asthma, COPD, hypertension, hyperlipidemia, coronary artery disease, dysrhythmia, esophageal disease, peptic ulcer, hepatitis B virus infection, chronic liver disease, and chronic kidney disease), and higher proportions of medication usage (including NSAIDs, disease-modifying anti-rheumatic drugs (DMARDs), corticosteroids, PPIs, H2 receptor antagonists, aspirin, and oral antihypertensive drugs) when compared with non-AS group (Table 2).

**Table 1:** Characteristics among groups.

	**Control** ***n* = 8,840**	**Patients with AS** ***n* = 2,210**	***p*-value**
Age at index date			1.0000
<20	524 (5.93%)	131 (5.93%)	
20–39	4,116 (46.56%)	1,029 (46.56%)	
40–59	2,880 (32.58%)	720 (32.58%)	
>= 60	1,320 (14.93%)	330 (14.93%)	
Sex			1.0000
Female	3,128 (35.38%)	782 (35.38%)	
Male	5,712 (64.62%)	1,428 (64.62%)	
Urbanization			0.7017
Urban	5439 (61.53%)	1356 (61.36%)	
Sub-urban	2626 (29.71%)	648 (29.32%)	
Rural	775 (8.77%)	206 (9.32%)	
Low income	59 (0.67%)	5 (0.23%)	0.0145
Length of hospital stay			<0.0001
0	7811 (88.36%)	1855 (83.94%)	
1–6	621 (7.02%)	197 (8.91%)	
7–13	211 (2.39%)	96 (4.34%)	
>= 14	197 (2.23%)	62 (2.81%)	
Co-morbidities			
Other rheumatic diseases	159 (1.80%)	202 (9.14%)	<0.0001
Thyroid disorders	152 (1.72%)	81 (3.67%)	<0.0001
Asthma	329 (3.72%)	119 (5.38%)	0.0004
COPD	534 (6.04%)	202 (9.14%)	<0.0001
Hypertension	1225(13.86%)	379(17.15%)	<0.0001
Diabetes mellitus	613 (6.93%)	171 (7.74%)	0.1884
Hyperlipidemia	821 (9.29%)	285 (12.90%)	<0.0001
Coronary artery disease	442 (5.00%)	171 (7.74%)	<0.0001
Dysrhythmia	237 (2.68%)	108 (4.89%)	<0.0001
Heart failure	93 (1.05%)	30 (1.36%)	0.2209
Cerebrovascular accident	278 (3.14%)	87 (3.94%)	0.0625
Overweight or obesity	86 (0.97%)	13 (0.59%)	0.0861
Esophageal disease	396 (4.48%)	226 (10.23%)	<0.0001
Peptic ulcer	724 (8.19%)	357 (16.15%)	<0.0001
Hepatitis B virus infection	201 (2.27%)	77 (3.48%)	0.0012
Hepatitis C virus infection	61 (0.69%)	22 (1.00%)	0.1369
Chronic liver disease	621 (7.02%)	265 (11.99%)	<0.0001
Chronic kidney disease	175 (1.98%)	73 (3.30%)	0.0002
Other respiratory abnormalities	82 (0.93%)	33 (1.49%)	0.0191
Hypertrophic tonsillitis	2 (0.02%)	4 (0.18%)	0.0043
Adenoid hypertrophy	1 (0.01%)	1 (0.05%)	0.2888
Chronic rhinitis	433 (4.9%)	164 (7.42%)	<0.0001
Hypertrophy of nasal turbinates	573 (6.48%)	225 (10.18%)	<0.0001
Nasal septum derivation	71 (0.8%)	40 (1.81%)	<0.0001

**Table 2:** Medication among groups within 180 days before or after index date.

	**Control**	**Patients with AS**	***p*-value**
**NSAIDs**	4111 (46.50%)	2032 (91.95%)	<0.0001
Aspirin	522 (5.90%)	188 (8.51%)	<0.0001
Indomethacin	161 (1.82%)	177 (8.01%)	<0.0001
Piroxicam	298 (3.37%)	293 (13.26%)	<0.0001
Diclofenac	2224 (25.16%)	1365 (61.76%)	<0.0001
Nabumetone	20 (0.23%)	57 (2.58%)	<0.0001
Naproxen	267 (3.02%)	228 (10.32%)	<0.0001
Sulindac	133 (1.5%)	308 (13.94%)	<0.0001
Tiaprofenic acid	81 (0.92%)	143 (6.47%)	<0.0001
Tenoxicam	46 (0.52%)	56 (2.53%)	<0.0001
Ibuprofen	1204 (13.62%)	502 (22.71%)	<0.0001
Celecoxib	76 (0.86%)	463 (20.95%)	<0.0001
Mefenamic acid	1495 (16.91%)	560 (25.34%)	<0.0001
Ketorolac	283 (3.2%)	274 (12.4%)	<0.0001
Meloxicam	107 (1.21%)	410 (18.55%)	<0.0001
**DMARDs**	112 (1.27%)	752 (34.03%)	<0.0001
Hydroxychloroquine	17 (0.19%)	89 (4.03%)	<0.0001
Leflunomide	0 (0.00%)	2 (0.09%)	0.0047
Methotrexate	10 (0.11%)	41 (1.86%)	<0.0001
Azathioprine	5 (0.06%)	9 (0.41%)	<0.0001
Ciclosporin	2 (0.02%)	5 (0.23%)	0.0007
Sulfasalazine	9 (0.10%)	680 (30.77%)	<0.0001
Minocycline	81 (0.92%)	29 (1.31%)	0.0936
Corticosteroids	1147 (12.98%)	632 (28.60%)	<0.0001
PPI	169 (1.91%)	109 (4.93%)	<0.0001
H2 receptor antagonist	1063 (12.02%)	475 (21.49%)	<0.0001
Aspirin	522 (5.90%)	188 (8.51%)	<0.0001
Oral antihypertensive drugs	1263 (14.29%)	461 (20.86%)	<0.0001
Oral hypoglycemic agents	387 (4.38%)	97 (4.39%)	0.9815
Statin	325 (3.68%)	97 (4.39%)	0.1179

*Oral antihypertensive drugs, including Alpha-blockers, Beta- blockers, CCBs, ACEI, ARBs*.

*Oral hypoglycemic agents, including Biguanides, Sulfonylureas, Alpha glucosidase inhibitors, Thiazolidinediones*.

Table 3 shows the HRs of OSA. On univariate modeling, the crude HR was 3.031 (95% C.I. = 1.888–4.865) in patients with AS. On multivariate modeling, the adjusted HR (aHR) was 2.794 (95% C.I. = 1.705–4.578) in patients with AS. The significantly associated risk factors were male gender (aHR = 2.174, 95% C.I. = 1.202–3.9), asthma (aHR = 2.371, 95% C.I. = 1.027–5.472), esophageal disease (aHR = 2.469, 95% C.I. = 1.185–5.143), and hepatitis B viral infection (aHR = 3.523, 95% C.I. = 1.401–8.857). NSAIDs use (aHR = 1.922, 95% C.I. = 0.947–3.899) was borderline significantly associated with OSA.

**Table 3:** Estimation the hazard ratio of OSA by using Cox proportional hazard regression.

	**Univariate modeling**		**Multivariate modeling**	
	**HR**	**95% C.I**.	**aHR**	**95% C.I**.
Exposure of AS (ref: non AS)				
**AS patient**	3.031	1.888–4.865	2.794	1.705–4.578
Age at index date (ref: 20-39)				
<20	0.559	0.134–2.345	0.598	0.142–2.519
40–59	1.751	1.063–2.884	1.629	0.943–2.814
>= 60	0.754	0.313–1.817	0.593	0.211–1.669
Sex (ref: Female)				
**Male**	1.957	1.106–3.464	2.174	1.202–3.931
Urbanization (ref: Urban)				
Sub-urban	1.224	0.746–2.008	1.208	0.733–1.991
Rural	0.495	0.153–1.597	0.433	0.132–1.421
Length of hospital stay (ref: 0)				
1–6	1.618	0.773–3.389	1.158	0.531–2.526
7–13	0.573	0.079–4.137	0.447	0.059–3.364
>= 14	2.414	0.756–7.709	2.03	0.599–6.881
Co-morbidities				
Other rheumatic diseases	0.438	0.061–3.153	0.245	0.033–1.838
Thyroid disorders	1.568	0.384–6.398	0.826	0.165–4.142
**Asthma**	2.810	1.287–6.135	2.371	1.027–5.472
COPD	2.089	1.038–4.206	1.461	0.676–3.16
Hypertension	1.708	0.951–3.069	1.742	0.839–3.616
Diabetes mellitus	1.438	0.622–3.321	1.24	0.48–3.2
Hyperlipidemia	1.507	0.749–3.036	0.837	0.361–1.941
Coronary artery disease	2.120	0.971–4.628	1.99	0.787–5.029
Dysrhythmia	1.523	0.479–4.842	0.906	0.261–3.143
Heart failure	–	–	–	–
Cerebrovascular accident	0.483	0.067–3.476	0.285	0.037–2.194
**Esophageal disease**	4.243	2.226–8.089	2.469	1.185–5.143
Peptic ulcer	2.121	1.161–3.874	1.203	0.598–2.419
**Hepatitis B virus infection**	3.907	1.692–9.025	3.523	1.401–8.857
Hepatitis C virus infection	–	–	–	–
Chronic liver disease	2.218	1.191–4.130	1.201	0.589–2.452
Chronic kidney disease	–	–	–	–
786.09	1.82	0.253–13.107	1.218	0.156–9.492
Hypertrophic tonsillitis	–	–		
Adenoid hypertrophy	–	–		
Chronic rhinitis	2.132	0.976–4.656	1.518	0.669–3.445
Hypertrophy of nasal turbinates	1.63	0.746–3.56	1.21	0.531–2.754
Nasal septum	1.399	0.195–10.057	0.656	0.085–5.057
Medications				
**NSAIDs**	1.625	0.918–2.877	1.922	0.947–3.899
DMARDs	–	–	–	–
Corticosteroids	1.416	0.516–3.886	0.754	0.234–2.426
PPI	4.403	1.079–17.967	2.262	0.413–12.382
H2 Receptor	2.367	0.953–5.880	1.344	0.432–4.185
Aspirin	0.877	0.122–6.312	0.712	0.087–5.852
Antihypertensive drugs	0.742	0.182–3.027	0.343	0.066–1.784
Antihyperglycemic agents	1.555	0.216–11.196	1.108	0.12–10.258
Statin	1.662	0.231–11.971	1.526	0.163–14.292

The incidence rates (per 100,000 person months) of OSA were 7.54 (95% C.I. = 5.53–10.28) and 22.84 (95% C.I. = 15.97–32.68) for control and AS groups, respectively. We conducted 4 different proportional hazard models to examine the stability of aHR. The aHRs did not show large variance with a range from 2.718 (95% C.I. = 1.670–4.423) to 3.036 (95% C.I. = 1.891–4.875) (Table 4).

**Table 4:** Time to event analysis.

	**Control**	**Patients with AS**	***p*-value**
N	8,840	2,210	
Follow up person months	530518	131318	
Event of OSA	40	30	
Incidence rate^*^ (95% C.I.)	7.54 (5.53–10.28)	22.84 (15.97–32.68)	
Model 1: Crude hazard ratio (95% C.I.)	Reference	3.031 (1.888–4.865)	<0.0001
Model 2: aHR (95% C.I.)	Reference	3.036 (1.891–4.875)	<0.0001
Model 3: aHR (95% C.I.)	Reference	2.718 (1.670–4.423)	<0.0001
Model 4: aHR (95% C.I.)	Reference	2.826 (1.727–4.625)	<0.0001

* *per 100,000 person months*.

*Model 2: adjusted for demographic variables, including sex, age, and urbanization at baseline*.

*Model 3: adjusted for demographic variables, length of hospital stay, and co-morbidities at baseline*.

*Model 4: adjusted for demographic variables, length of hospital stay, co-morbidities, and co-medications at baseline*.

**Figure 2:**
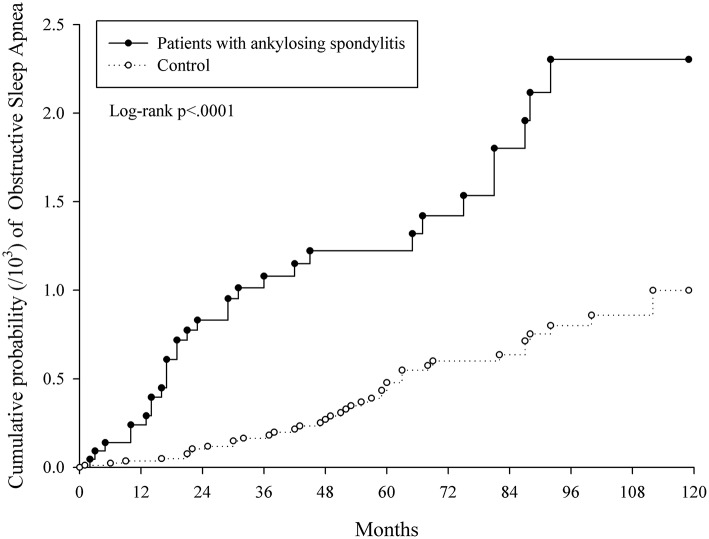
The cumulative proportions of OSA in both AS and non-AS groups.

Figure 2 indicates the cumulative proportions of OSA in both AS and non-AS groups. Higher cumulative proportion in AS group was observed and the log-rank test *p* was <0.0001. According to the slope of Kaplan-Meier curves and test for proportional assumption, the risk of OSA in AS is time dependent. Therefore, landmark analysis (Table 5) was conducted to explore the risk of OSA during specific follow-up periods. The aHR was 7.919 (95% C.I. = 3.169–19.792) in AS group at 0–24 months from index date and decreased to 1.816 (95% C.I. = 0.944–3.494) at ≥ 24 months from index date.

**Table 5:** Landmark analysis.

	**Control**			**AS patients**			
	**Person months**	**Event**	Incidence* (95% C.I.)	**Person months**	**Event**	Incidence* (95% C.I.)	**aHR‡ (95% C.I.)**
**Follow up time interval**							
Index date to 24 months	188,613	8	4.24 (2.12–8.48)	46,810	16	34.18 (20.94–55.79)	7.919 (3.169–19.792)
>24 months	341,905	32	9.36 (6.62–13.23)	84,508	14	16.57 (9.81–27.98)	1.816 (0.944–3.494)
p for interaction							0.0087

Table 6 shows the results of subgroup analyses. On sex stratified analysis (p for interaction was 0.5428), aHRs were 4.533 (95% C.I. = 1.441–14.262) for females and 2.672 (95% C.I. = 1.522–4.692) for males. On age stratified analysis (p for interaction was 0.7562), aHRs were 0.719 (95% C.I. = 0.015–35.162) for those <20, 1.847 (95% C.I. = 0.830–4.108) for those 20–39, 3.913 (95% C.I. = 1.890–8.102) for those 40–59, and 3.930 (95% C.I. = 0.665–23.234) for those ≥60.

**Table 6:** Subgroup analysis.

	**Control**			**AS patients**			
	**Person months**	**Event**	Incidence^*^ (95% C.I.)	**Person months**	**Event**	Incidence^*^ (95% C.I.)	**aHR‡ (95% C.I.)**
**Sex subgroups**							
Female	184,568	8	4.33 (2.17–8.67)	46,172	7	15.16 (7.23–31.80)	4.533 (1.441–14.262)
Male	345,950	32	9.25 (6.54–13.08)	85,146	23	27.01 (17.95–40.65)	2.672 (1.522–4.692)
p for interaction							0.5428
**Age subgroups**							
<20	31,924	1	3.13 (0.44–22.24)	8,016	1	12.47 (1.76–88.56)	0.719 (0.015–35.162)
20-39	258,338	19	7.35 (4.69–11.53)	64,600	10	15.48 (8.33–28.77)	1.847 (0.830–4.108)
40-59	168,657	17	10.08 (6.27–16.21)	41,679	16	38.39 (23.52–62.66)	3.913 (1.890–8.102)
>= 60	71,599	3	4.19 (1.35–12.99)	17,023	3	17.62 (5.68–54.65)	3.930 (0.665–23.234)
p for interaction							0.7562

* *per 100,000 person months*.

‡ *Adjust for variables, including age, gender, residential urbanization, length of hospital stay, comorbidity, and drug use at baseline*.

## Discussion

To the best of our knowledge, this is the first retrospective cohort study with long term follow up using nationwide population-based data to investigate the OSA risk associated with AS. The nationwide study demonstrated that AS patients were at an increased risk (2.794-fold greater)of developing OSA compared with non-AS controls. This association was more pronounced within the first two years of diagnosis of AS. The interaction terms for sex and age were insignificant, suggesting that the magnitude of association did not vary significantly between males and females or across age groups (Please deleted). The results of this study showed a 2.794-fold greater risk of subsequent development of OSA among AS patients than the general population. Furthermore, stratified analyses revealed significant effects for both genders and the 40–59 age group. Although the AS group had a significantly higher rate of comorbid diseases compared to the non-AS group, AS remained an independent risk factor for developing OSA after adjusting for gender, age, comorbidities and co-medications.

There are four significant findings of this study. First, it is currently the only large cohort study to investigate the association between AS and the subsequent development of OSA. The Taiwan NHI Research Database is one of the largest nationwide population databases in the world, covering approximately 23 million residents in Taiwan (35, 36). The study cohorts were large enough to observe the risk variations among subgroups and inform on the incidences, treatments, correlates, and associations of disease, as well as on the patterns of health care utilization. The major advantages include enormous sample size and lack of selection or participation bias (36). Second, we performed concise subgroup analyses to illustrate the interrelationships of gender, age, comorbidities, and medications. This can help to identify and appropriately monitor the high-risk groups of AS patients, such as male subjects and those aged 40–59. Third, the validity of the findings was enhanced by unbiased subject selection and strict criteria for the diagnosis of OSA. Fourth, significant risk of developing OSA (aHR was 7.919 with 95% C.I. = 3.169–19.792) was noted in the first 2 years after diagnosis of AS. We speculated that the disease activity of AS is controlled by medications and physical therapy, leading to a decrease in the associated risk of OSA.

The underlying mechanism of the relationship between AS and OSA remains largely unclear. The possible mechanisms of OSA in AS patients include restriction of the oropharyngeal airway due to temporomandibular joint involvement, pharyngeal, and tracheal compression by cervical spine disease, and restrictive pulmonary disease (33, 34). There is a possible role for cytokines in the regulation of sleep in patients with systemic inflammatory disorders (37, 38).

Our present study also identified that asthma, esophageal disease, hepatitis B infection were associated with a greater risk of OSA after multivariable adjustment. In a small study, the prevalence of OSA in asthma patients was 46% among 50 patients with age range 30–68 years. Of them, 12% patients had mild OSA, 14% had moderate while 20% were having severe OSA. Most common medical comorbidities in patients with OSA was GERD (78.26%) followed by allergic rhinitis (56%). OSA is not uncommon in asthma patients (39). In addition, Wisconsin Sleep Cohort Study had shown asthma is associated with the development of OSA. The adjusted relative risk of developing OSA was 1.39 (95% CI, 1.06–1.82) after adjusting for age, gender, baseline and change in body mass index, and other factors. Therefore, further studies exploring the underlying biological mechanisms and the value of periodic OSA evaluation in patients with asthma are warranted (40). The causative relationship between GERD and OSA remains an area of controversy (41). A previous study had shown an association between non-erosive gastroesophageal reflux disease with increased risk of OSA in Korean Population (42). More patients in the GERD group (28.2%) had higher risk for obstructive sleep apnea than healthy controls (20.4%, *P* = 0.036). Nocturnal GERD was related to high risk for OSA in non-erosive disease patients (OR, 2.97; *P* = 0.019), but not in erosive disease patients (42). The reflux of acids may result in spasms of the vocal cords that can then lead to sleep apnea (43).

Several limitations should be considered when interpreting the findings of this study. First, information on potential confounding factors, such as body mass index, family history, and drinking and smoking habits was unavailable. Smoking increases the risk of OSA or at least aggravates preexisting symptoms. However, we used COPD as a proxy variable for cigarette smoking, based on the accepted methodology of several previous studies (44–46). It is worthy of considering obesity as the covariate in the multivariable analysis, as it is one of the well-established risk factors for OSA. Although some coding such as 278.00 for obesity and 278.01 for morbid obesity might be recorded in the LHIRD database, these coding was not accurate enough. Very few Taiwanese physicians used these two codes. Instead, we have included some obesity associated comorbidities such as diabetes mellitus and hyperlipidemia as covariates in the regression model. Second, NHIRD did not provide detailed information on the severity of AS or OSA, and it was therefore not possible to demonstrate the dose–response relationship between AS and OSA. Third, an important issue is the lack of relevant variables such as polysomnography results, image reports, physical examination findings (as neck circumference, occiput-to-wall distance, schober's test and chest expansion), and information about disease activity were unavailable in the current insurance databases. Results from polysomnography, the gold standard for the diagnosis of OSA, were not available in the LHIRD. Our findings, therefore, should be interpreted with caution given the above mentioned methodological flaws.

## Conclusion

This 11-year population-based cohort study demonstrated a higher risk of OSA in patients with AS, among both genders and those aged 40–59. The risk was highest within the first 2 years of diagnosis of AS. Further studies are recommended to clarify the underlying biological mechanisms of these associations. It is important to evaluate sleep quality and quantity for patients with AS to detect the occurrence of OSA and to reduce further complications.

## Data Availability Statement

All datasets generated for this study are included in the article/supplementary material.

## Author Contributions

C-HT, YM-H, and JW: study conception and design and writing of the paper. J-YH and JW: acquisition of data. C-HT, YM-H, JW, H-HH, and J-YH: analysis and interpretation of data. All authors were involved in drafting, revising this manuscript, and approved the final version for publication.

### Conflict of Interest

The authors declare that the research was conducted in the absence of any commercial or financial relationships that could be construed as a potential conflict of interest.
